# The epilepsy phenotype of *ST3GAL3*‐related developmental and epileptic encephalopathy

**DOI:** 10.1002/epi4.12747

**Published:** 2023-04-24

**Authors:** Robyn Whitney, Puneet Jain, Rajesh RamachandranNair, Kevin C. Jones, Hassan Kiani, Mark Tarnopolsky, Brandon Meaney

**Affiliations:** ^1^ Division of Neurology Department of Paediatrics McMaster University Hamilton Ontario Canada; ^2^ Epilepsy Program Division of Neurology Department of Paediatrics Hospital for Sick Children University of Toronto Toronto Ontario Canada; ^3^ Division of Neuromuscular and Neurometabolic Disorders Department of Paediatrics McMaster University Hamilton Ontario Canada

**Keywords:** congenital disorder of glycosylation, developmental and epileptic encephalopathy, genetics, genotype, ST3GAL3

## Abstract

**Objective:**

*ST3GAL3*‐related developmental and epileptic encephalopathy (DEE‐15) is an autosomal recessive condition characterized by intellectual disability, language and motor impairments, behavioral difficulties, stereotypies, and epilepsy. Only a few cases have been reported, and the epilepsy phenotype is not fully elucidated.

**Methods:**

A retrospective chart review of two siblings with *ST3GAL3*‐related DEE was completed. In addition, we reviewed all published cases of *ST3GAL3‐*related congenital disorder of glycosylation.

**Results:**

Two brothers presented with global developmental delay, motor and language impairment, hypotonia, and childhood‐onset seizures. Seizures started between 2.5 and 5 years and had tonic components. Both siblings had prolonged periods of seizure freedom on carbamazepine. Tremor was present in the younger sibling. Whole exome sequencing revealed two novel pathogenic variants in *ST3GAL3*, (a) c.302del, p.Phe102Serfs*34 and (b) c.781C>T, p.Arg261*, which were inherited *in trans*. Magnetic resonance imaging showed T2 hyperintensities and restricted diffusion in the brainstem and middle cerebellar peduncle in the older sibling, also described in two reported cases. A review of the literature revealed 24 cases of *ST3GAL3*‐related CDG. Twelve cases had information about seizures, and epilepsy was diagnosed in 8 (67%). The median age of seizure onset was 5.5 months. Epileptic spasms were most common (67%). Four children were diagnosed with Infantile Epileptic Spasms syndrome and Lennox Gastaut syndrome (57%). Most children (n = 6, 75%) had seizures despite anti‐seizure medication treatment.

**Significance:**

Seizures related to *ST3GAL3*‐related DEE often occur in infancy and may present as epileptic spasms. However, seizure onset may also occur outside of infancy with mixed seizure types and show good response to treatment with prolonged seizure freedom. Tremor may also be uniquely observed in this condition.

**Key Points**

- *ST3GAL3*‐related developmental and epileptic encephalopathy (DEE‐15) is a rare autosomal recessive condition with few cases reported
- The epilepsy phenotype may include infantile‐onset epileptic spasms and evolution to Lennox Gastaut syndrome and medically refractory epilepsy
- Later‐onset childhood seizures which respond to anti‐seizure medication (ASMs) treatment with prolonged periods of seizure freedom may also occur
- The EEG findings are variable; neuroimaging is generally normal, but may show T2‐magnetic resonance imaging hyperintensity within the brainstem and cerebellar peduncles
- There is limited data to support the use of specific ASMs in *ST3GAL3*‐related developmental and epileptic encephalopathy, although this may depend on the seizure/epilepsy type

## 1 INTRODUCTION

Our understanding of genetic causes of epilepsy and advancements in genetic testing have changed how we care for patients with epilepsy and their families.^1^, ^2^, ^3^ A growing number of genetic variants are associated with epilepsy, and precision therapies exist for some genetic forms of epilepsy. Up to 50% of epilepsies may now have an identifiable genetic cause, and several disease mechanisms are implicated.^1^, ^2^, ^3^, ^4^ Monogenic causes of epilepsy have contributed the most to our understanding of rare developmental and epileptic encephalopathies (DEEs).^1^, ^2^ In most cases, patients harbor a de novo dominant pathogenic variant.^1^ However, other inheritance patterns may occur in some cases, such as somatic mosaicism and autosomal recessive inheritance.^1^, ^4^ There are a growing number of rare autosomal recessive genes that may result in DEE phenotypes, one of which includes biallelic variants in *ST3GAL3*.^5^, ^6^, ^7^, ^8^, ^9^, ^10^

The *ST3GAL3* gene encodes for the Golgi membrane enzyme, beta‐galactosidase‐alpha‐2,3 sialytransferase‐III (ST3GAL3), which is highly expressed in the developing brain.^1^, ^10^, ^11^, ^12^, ^13^ ST3GAL3 is involved in the formation of sialyl epitopes on glycoproteins, which help to determine the functional specificity of glycans, and are also important in brain function and development.^13^ Moreover, sialyl epitopes formed on glycoproteins regulate cell–cell interactions.^11^ Loss of function variants in *ST3GAL3* can cause non‐syndromic intellectual disability (ID) and may also cause a rare form of autosomal recessive developmental and epileptic encephalopathy (DEE‐15).^5^, ^6^, ^7^, ^8^, ^9^, ^10^, ^11^, ^12^, ^13^ Only twenty‐four individuals with *ST3GAL3*‐related congenital disorder of glycosylation (CDG) have been described in the literature. The phenotype described includes moderate to severe ID, language and motor impairment, behavioral disorders, hypotonia, and in some cases, epilepsy.^5^, ^6^, ^7^, ^8^, ^9^

Infantile epileptic spasms syndrome (IESS) and Lennox Gastaut syndrome (LGS) have been described in some cases of *ST3GAL3‐*related DEE.^8^ However, the electroclinical phenotype of *ST3GAL3*‐related DEE has not been fully elucidated. Herein, we report two siblings with *ST3GAL3*‐related DEE with novel pathogenic variants in *ST3GAL3* and further add to the phenotypic spectrum of epilepsy in this condition. A comprehensive review of the literature on *ST3GAL3*‐related CDG with a focus on epilepsy is also presented.

## 2 MATERIALS AND METHODS

A retrospective chart review was completed after ethics approval and consent was obtained from the patients' caregivers as per the local research ethics guidelines. Abstracted data included: the age of onset of epilepsy, seizure types, developmental history, neurological comorbidities, electroencephalogram findings (EEG), neuroimaging findings, genetic results, and response to different treatments. The American College of Medical Genetics (ACMG) criteria were used to classify the *ST3GAL3* variants (Table 1).^14^ We also conducted a focused, comprehensive narrative literature review of previously reported cases of *ST3GAL3*‐related CDG. The databases PubMed, Google Scholar, and Scopus were searched until March 1, 2023, using the following terms: *ST3GAL3*, *ST3GAL3* epilepsy, *ST3GAL3* seizures, and *ST3GAL3* DEE. Publication citations were screened to look for additional cases and duplicate cases. All cases of *ST3GAL3‐*related CDG were then collected, and data were abstracted by RW and PJ (Table 2). Variables of interest collected included: age of seizure onset, seizure types, International League Against Epilepsy (ILAE) epilepsy syndrome, comorbidities, genetic results, EEG findings, magnetic resonance imaging (MRI) findings, and response to different treatments (Table 2).

**TABLE 1:** Novel variants identified in *ST3GAL3*.

Variant	ACMG criteria	Inheritance/novel or previously reported	Classification
*ST3GAL3*, NM_006279.3: c.302del, p.Phe102Serfs*34	PVS1‐Very strong PM1, PM2, PM3—moderate	Inherited (Paternal) Novel	Pathogenic
*ST3GAL3*, NM_006279.3: c.781C>T, p.Arg261*	PVS1—Very strong PM1, PM2, PM3‐ moderate	Inherited (Maternal) Novel	Pathogenic

**TABLE 2:** Previously reported cases of ST3GAL3‐related CDG.

Case:	Age/sex (in years)	Age of seizure onset (mo)	Seizure types	ILAE epilepsy syndrome	Comorbidity	Genetics	Zygosity	EEG	MRI	Treatment	References
1	12, M	36	Few seizures no details	–	ID, IQ < 40 ASD Aggression Hypotonia Repetitive behaviors Dysarthria Dyspraxia Poor visual tracking	c.936 + 1delG	HMZ	–	Normal	VPA: Sz freedom	[5]
2	35, F	–	–	–	ID, IQ 40	c.38C>A, p.Ala13Asp	HMZ	–	–	–	[9]
3	34, M	–	–	–	ID, IQ 25	c.38C>A, p.Ala13Asp	HMZ	–	–	–	[9]
4	40, M	–	–	–	ID, IQ 30	c.38C>A, p.Ala13Asp	HMZ	–	–	–	[9]
5	23, M	–	–	–	ID, IQ 35	c.38C>A, p.Ala13Asp	HMZ	–	–	–	[9]
6	17, M	–	–	–	ID, IQ 40 Severe kyphosis	c.38C>A, p.Ala13Asp	HMZ	–	–	–	[9]
7	22, F	–	–	–	ID, IQ 45	c.38C>A, p.Ala13Asp	HMZ	–	–	–	[9]
8	18, F	–	–	–	ID, IQ 55	c.38C>A, p.Ala13Asp	HMZ	–	–	–	[9]
9	15, F	–	–	–	ID, IQ 40	c.38C>A, p.Ala13Asp	HMZ	–	–	–	[9]
10	34, M	–	–	–	ID, IQ 30 Brachydactyly	c.1108G>T, p.Asp370Tyr	HMZ	–	–	–	[9]
11	17, M	–	–	–	ID, IQ 40	c.1108G>T, p.Asp370Tyr	HMZ	–	–	–	[9]
12	16, F	–	–	–	ID, IQ 40	c.1108G>T, p.Asp370Tyr	HMZ	–	–	–	9
13	4, M	–	–	–	ID, IQ 50	c.1108G>T, p.Asp370Tyr	HMZ	–	–	–	[9]
14	16, F	3	Rare atonic Previous spasms	IESS‐ 8 mo LGS—2 y	Severe ID Non‐verbal	c.958G>C, p.Ala320Pro	HMZ	Hyps	Normal	VBG, ACTH, CLB, VPA, LEV—ineffective	[8]
15	2, M	7	Atonic, tonic, myoclonic Previous spasms	IESS‐ 1 y LGS—2 y	Severe ID Non‐verbal Poor eye contact Irritability Non‐ambulatory	c.958G>C, p.Ala320Pro	HMZ	Hyps	Normal	VBG‐effective ACTH, TPM, CLB, LEV, RUF‐ all ineffective	[8]
16	10, M	4	Rare GTC Previous spasms	IESSS‐ 7 mo LGS—3 y	Severe ID Non‐verbal	c.958G>C, p.Ala320Pro	HMZ	Hyps	Normal	VBG‐effective ACTH, CBZ, CLB, LEV, RUF‐ all ineffective	[8]
17	4, M	3	Rare focal clonic Previous spasms	IESSS‐ 6 mo LGS—4 y	Severe ID Non‐verbal	c.958G>C, p.Ala320Pro	HMZ	Hyps	Normal	VBG‐effective VPA, CBZ, CLB, LEV‐all ineffective	[8]
18	1.5, M	7	Daily events of eye deviation and behavioral arrest Clonus of head and upper limbs	DEE not specified	Motor delay Hypotonia Limited language Posterior plagiocephaly	c.660C>A, p.Tyr220Ter	HMZ	Diffuse IEDs sleep Focal frontal sharp waves	DWI restriction PAG, posterior pons, mesencephalic region, middle cerebellar peduncles; corresponding high T2	VPA, ETX‐ineffective	[6]
19	1.5, F	8	Events of eye deviation and behavioral arrest	DEE not specified	Motor delay Hyperexcitable Hypotonia Stereotypies Limited language	c.660C>A, p.Tyr220Ter	HMZ	Diffuse IEDs	DWI restriction PAG, posterior pons, mesencephalic region, middle cerebellar peduncles; corresponding high T2	VPA, ETX‐ seizures controlled	[6]
20	31, F	None	None	None	ID, IQ < 40 No ADLs Walking difficulty Frequent falls Loss of acquired skills Aggression Poor comprehension Some speech Chronic lung disease Neonatal jaundice	c.704C>T, p.Thr235Met	HMZ	Normal	Normal	None	[7]
21	27, M	None	None	None	ID <40 No ADLs Walking difficulty Frequent falls Loss of acquired skills Aggression Poor comprehension Some speech Chronic lung disease Neonatal jaundice	c.704C>T, p.Thr235Met	HMZ	–	–	–	[7]
22	22, F *Deceased*	None	None	None	ID <40 No ADLs Walking difficulty Frequent falls Loss of acquired skills Aggression Poor comprehension No speech Neonatal jaundice	c.704C>T, p.Thr235Met (assumed)	HMZ	–	–	–	[7]
23	29, F *Deceased*	None	None	None	ID <40 Walking difficulty Frequent falls Loss of acquired skills Aggression Poor comprehension Some speech Chronic lung disease Neonatal jaundice	c.704C>T, p.Thr235Met (assumed)	HMZ	–	–	–	[7]
24	2, F	4	–	DEE not specified, drug resistant epilepsy		c.846delT, p.E283RfsTer40	HMZ	–	–	Drug resistant	[10]

Abbreviations: – = information not available; ACTH, adrenocorticotrophic hormone; ADLs, activities of daily living; ASD, autism spectrum disorder; CBZ, carbamazepine; CDG, congenital disorder of glycosylation; CLB, clobazam; DEE, developmental and epileptic encephalopathy; DWI, diffusion‐weighted imaging; ETX, ethosuximide; GDD, global developmental delay; GTC, generalized tonic–clonic; HMZ, homozygous; Hyps, hypsarrhythmia; ID, intellectual disability; IEDs, interictal epileptiform discharges; IESS, infantile epileptic spasms syndrome; IQ, intelligence quotient; LEV, levetiracetam; LGS, Lennox Gastaut syndrome; mo, months; PAG, periaqueductal gray; RUF, rufinamide; Sz, seizure; TPM, topiramate; V, valproic acid.

## 3 RESULTS

### 3.1 Case 1

The index case is a 12‐year‐old male born prematurely at 29 weeks' gestation via Caesarian section due to placenta previa. Parents were non‐consanguineous. A prolonged neonatal intensive care stay complicated the neonatal course due to prematurity. However, there were no neurological comorbidities. At 9 months of age, global developmental delay was documented (GDD) as well as hypotonia. The family history was non‐contributory. At 2.5 years of age, MRI of the brain was completed. The MRI showed symmetrical abnormal high T2 signal with corresponding diffusion restriction involving the superior olivary nuclei, middle cerebellar peduncles, and dentate nuclei. Magnetic resonance spectroscopy was normal. However, these findings were felt to be non‐specific but could represent a metabolic disorder. Although an extensive metabolic workup was negative. He continued to have global developmental delays throughout childhood, and there were some recurrent infections.

**FIGURE 1:**
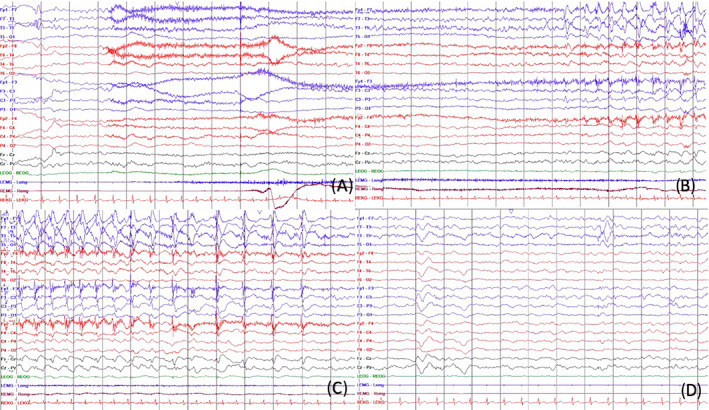
Ictal and interictal electroencephalogram findings. EEG epochs A to C: sensitivity 20 uV/mm, timebase 30 mm/s, high‐frequency filter = 70 Hz, low‐frequency filter = 1 Hz. Epochs A to C represent a focal asymmetric tonic seizure followed by right‐sided extremity clonic movements in the older sibling's case (case 1). Ictal onset was from the left temporal head region, which is then followed by diffuse attenuation of the EEG and then by focal spike and slow wave over the left temporal head regions and then the left parasagittal head regions with fields to the right. Epoch D, sensitivity 20 uV/mm, timebase 30 mm/s, high‐frequency filter = 70 Hz, low‐frequency filter = 1 Hz. Epoch D represents typical interictal activity seen throughout the EEG and shows focal spike and slow wave maximal over the left temporal head regions; here in NREM sleep.

At 5 years of age, the child developed focal motor seizures with impaired awareness, associated with asymmetric tonic posturing of the upper extremities which could be followed by clonic movements of the right extremities, lasting around 1 minute. Prolonged video‐EEG at this time showed slow background activity for age (ie, in the theta range) and focal sharp and spike and slow waves over the left temporal head region. There were multiple asymmetric tonic followed by right clonic seizures captured during this recording with onset from the left frontotemporal head regions (Figure 1). MRI was repeated at the age of 5 years and showed similar findings as previously described. The child was initially trialed on valproate and clobazam, and neither were effective. Carbamazepine monotherapy was subsequently initiated and led to a prolonged period of seizure freedom for over 5 years, except for one recent seizure in the context of illness. The carbamazepine dose was increased after this seizure, and there has been no seizure recurrence.

A comprehensive epilepsy gene panel was completed after the onset of seizures and was non‐diagnostic. The child subsequently underwent whole exome sequencing, confirming two novel pathogenic variants in the *ST3GAL3* gene, NM_006279.3: c.302del, p.Phe102Serfs*34 and NM_006279.3:c.781C>T, p.Arg261*, which were inherited from each parent.

At the age of 12 years, the child is non‐verbal but is able to vocalize and uses an assistive communication device. He has strong receptive language. There is a history of some repetitive behaviors (ie, moving head backward repeatedly). He uses a wheelchair and walker, but he cannot walk unsupported. He is G‐tube fed and has a history of gastrointestinal reflux and strabismus.

### 3.2 Case 2

The younger brother of the index case is 10 years old. He was born at 38 weeks via repeat Caesarian section. The pregnancy, birth, and neonatal history were uncomplicated. Between 6 and 7 months of age, GDD was initially observed, as well as hypotonia. Focal motor seizures with preserved awareness began at the age of 2.5 years but were controlled with carbamazepine monotherapy for over 5 years. The seizures were characterized by asymmetric tonic posturing affecting the right arm and leg lasting up to 2 minutes. MRI of the brain was completed at 2.5 years and showed a single tiny focus of deep white matter signal abnormality in the left frontoparietal subcortical region, which was non‐specific in appearance. At 8 years of age, the previous seizures consisting of bilateral tonic stiffening of the upper extremities with unresponsiveness returned (ie, tonic seizures). These seizures lasted seconds and occurred multiple times per day. Awake routine EEG showed a mixture of alpha and theta frequencies, and no clear epileptiform discharges were seen. Carbamazepine was subsequently optimized, and levetiracetam was added, leading to seizure freedom. At the present time, the child has been seizure free for over a year.

At the age of 8 years, sequence analysis of the *ST3GAL3* gene revealed the same inherited pathogenic variants as his brother. At age 10, the child can walk independently but has a wheelchair and walker for long distances. He can walk up and down the stairs and uses sign language and an assistive communication device. He is social, with good receptive language. His neurological examination revealed tremor in the upper extremities as well as bilateral Achilles tendon spasticity.

### 3.3 Literature review

A total of 24 cases of *ST3GAL3*‐related CDG have been described in the literature from seven different families (Table 2).^5^, ^6^, ^7^, ^8^, ^9^, ^10^ Thirteen are male (54%), and the median age of reported cases is 17 years (IQR: 7, 28).^5^, ^6^, ^7^, ^8^, ^9^, ^10^ Twelve cases had information regarding the presence of seizures, and in eight cases (67%), epilepsy was diagnosed.^5^, ^6^, ^7^, ^8^, ^10^ The median age of seizure onset was 5.5 months (IQR: 3.5, 7.5). Six cases had information on observed seizure types. These included: epileptic spasms (n = 4, 67%), atonic seizures (n = 2, 33%), tonic seizures (n = 1, 17%), generalized tonic–clonic seizures (GTCs; n = 1, 17%), myoclonic seizures (n = 1, 17%), clonic seizures (n = 1, 17%), and focal motor seizures with impaired awareness (n = 2, 33%).^6^, ^8^ Information on the type of epilepsy syndrome was available in seven cases. Four children were diagnosed with IESS and subsequently LGS (57%).^8^ Three children were diagnosed with an unspecified DEE (43%).^6^, ^10^ EEG findings were available in seven cases and included findings such as hypsarrhythmia (n = 4, 57%), diffuse epileptiform discharges with or without frontal sharp waves (n = 2, 29%), and a normal recording (n = 1, 14%).^6^, ^7^, ^8^ MRI brain was available in eight cases and was normal in the majority (n = 6, 75%).^5^, ^7^, ^8^ However, in two cases, the MRI showed a high T2 signal and restricted diffusion in the periaqueductal gray, posterior pons, mesencephalic region, and middle cerebellar peduncles.^6^

A variety of different anti‐seizure medications (ASMs) were used to treat seizures and included: valproic acid (n = 4), vigabatrin (n = 4), adrenocorticotrophic hormone (n = 3), ethosuximide (n = 2), clobazam (n = 2), levetiracetam (n = 4), carbamazepine (n = 2), topiramate (n = 1), and rufinamide (n = 1).^5^, ^6^, ^7^, ^8^, ^10^ Two children had their seizures controlled with ASMs (25%), one with valproic acid monotherapy and one with valproic acid and ethosuximide.^5^, ^6^ The remaining children (n = 6, 75%), continued to have seizures, despite ASM therapy.^6^, ^8^, ^10^

Neurological comorbidities are summarized in Table 2 and included moderate to severe ID in 21 cases (91%); in two cases, there was motor delay and limited language, but no formal diagnosis of ID, and in one case, the presence of ID was unknown.^5^, ^6^, ^7^, ^8^, ^9^, ^10^ Other neurological comorbidities included: language impairments/non‐verbal status (n = 11, 46%), aggression/irritability (n = 6, 25%), motor delay/non‐ambulatory status (n = 3, 13%), frequent falls/walking difficulty (n = 4, 17%), hypotonia (n = 3, 13%), stereotypies/repetitive behaviors (n = 2, 8%), poor eye contact/visual tracking (n = 2, 8%), and dyspraxia (n = 1, 4%).^5^, ^6^, ^7^, ^8^, ^10^ Few systemic comorbidities were reported, such as kyphosis (n = 1, 4%) and chronic lung disease (n = 3, 13%).^7^, ^9^

## 4 DISCUSSION

In summary, we describe two additional siblings with *ST3GAL3*‐related DEE and a literature review of 24 previously reported cases of *ST3GAL3*‐related CDG (22 living, two deceased).^5^, ^6^, ^7^, ^8^, ^9^, ^10^ Our siblings showed some similarities to cases in the literature, including the presence of early onset GDD/ID, hypotonia, motor delays, walking difficulties, language impairment (ie, both were non‐verbal but had good comprehension), and seizures.^5^, ^6^, ^7^, ^8^, ^9^, ^10^ The older sibling had previously displayed some repetitive behaviors, which have also been described in a few cases.^5^, ^6^ Furthermore, the presence of a movement disorder in the form of tremor was observed in the younger brother. Although stereotypies have been reported, other movement disorders have not been documented previously.^6^ Systemic comorbidities in the form of feeding difficulties and childhood recurrent infections were observed in the older sibling, but not the younger. Previous cases have described rare systemic comorbidities, such as chronic lung disease in a few children with pathogenic variants in *ST3GAL3*.^7^ In contrast, other forms of CDG typically show more prominent multi‐systemic involvement.^12^, ^15^

Both siblings had seizures, consistent with several *ST3GAL3*‐related CDG cases described in the literature.^5^, ^6^, ^7^, ^8^, ^10^ However, some differences were observed in our siblings, who presented with seizures later than published cases, at the age of 5 and 2.5 years, respectively. The median age of seizure onset in the literature was 5.5 months, and only one case presented in childhood at the age of 3 years.^5^ In contrast to some cases in the literature, our siblings did not present with infantile epileptic spasms or evolve into the epilepsy syndrome, LGS.^8^ Infants typically presented with infantile epileptic spasms between the ages of 6‐12 months. While evolution to LGS occurred between the ages of 2 and 4 years.^8^ There was no clear ILAE epilepsy syndrome diagnosed in our sibling pair, either.^6^, ^10^ Furthermore, there was no clear history of regression in our siblings with seizure onset, which can occur with epileptic encephalopathies.

Seizure types reported in the literature were variable. They included a combination of generalized seizure types (ie, atonic, tonic, GTCs, myoclonic) and focal seizure types (ie, focal motor with impaired awareness).^6^, ^7^, ^8^ The most common seizures were epileptic spasms, starting between the ages of 3 and 7 months.^8^ Our sibling pair both had tonic components with their seizures, with the older sibling at times, also having a clonic component as described. Both children had prolonged periods of seizure freedom (ie, greater than 5 years) with carbamazepine, in contrast to the literature, where most children had medically refractory epilepsy and had trialed several ASMs.^6^, ^8^, ^10^ Seizures recurred in both siblings later in childhood but were subsequently controlled with ASM adjustment. Regarding the longitudinal outcome of seizures, most children continue to have seizures throughout childhood, as described, with seizures persisting into adolescence in some (ie, case 1, 14; Table 2).^5^, ^8^ Longitudinal follow‐up is needed into adulthood however to determine how the epilepsy evolves. ID was pervasive throughout childhood and adolescence with or without other neurological comorbidities.^5^, ^6^, ^7^, ^8^, ^9^, ^10^ Various ASMs were trialed in the literature, with different combinations, making it difficult to draw conclusions regarding which ASM is best for *ST3GAL3*‐related DEE. Two children were seizure‐free on valproic acid, either as monotherapy or in combination with ethosuximide.^5^, ^6^ Although it is difficult to draw precise conclusions regarding the most effective ASM treatment for *ST3GAL3*‐related DEE given the small numbers, this may depend on the seizure and epilepsy type. For example, in infants with infantile spasms epilepsy syndrome, vigabatrin may be effective, carbamazepine may be helpful for focal seizures and for children with a LGS phenotype or unspecified DEE, one may use medications like valproic acid.^5^, ^6^, ^8^ Larger case series are needed, however, to determine which ASMs are most effective for *ST3GAL3*‐related DEE.

The EEG was abnormal in most cases of *ST3GAL3*‐related DEE reported in the literature and showed findings such as generalized and focal epileptiform discharges, and in the case of IESS, hypsarrhythmia. The EEG recordings in our index case showed focal discharges (ie, left temporal) and slow background activity with seizure onset from the left frontotemporal head regions. The younger brother's EEG was normal in the awake state. Most published cases had normal neuroimaging of the brain. However, MRI was abnormal in both of our siblings. MRI showed high T2 signal and restricted diffusion in our index case's brainstem and middle cerebellar peduncles. These findings were also similarly described in twin siblings with *ST3GAL3*‐related DEE in the literature (Table 2).^6^ The MRI findings in our case were still present at the age of 5 years, although follow‐up imaging was not available for the two siblings in the literature. The significance of the brainstem changes is unclear and requires further investigation. It is possible that they could represent a neuroimaging finding specific to *ST3GAL3*‐related CDG. Although most types of CDG show cerebral and/or cerebellar atrophy on neuroimaging, these findings have not been associated with *ST3GAL3* pathogenic variants to date.^12^, ^15^

The variants reported in our sibling pairs (NM_006279.3:c.302del, p.Phe102Serfs*34 and NM_006279.3:c.781C>T, p.Arg261*) were novel and predicted to be pathogenic. Both variants are expected to result in a premature stop codon and absent protein due to nonsense‐mediated decay. Loss of function variants are the primary disease mechanism described in *ST3GAL3*‐related CDG.^5^, ^6^, ^7^, ^8^, ^9^, ^10^, ^11^, ^12^, ^13^ Seven other homozygous variants in *ST3GAL3* have been observed to be pathogenic in the literature.^5^, ^6^, ^7^, ^8^, ^9^, ^10^ Previous studies have suggested that the phenotype may be milder in cases with residual enzyme activity and result in non‐syndromic ID.^5^, ^6^, ^9^ The c.38C>A, p.Ala13Asp variant, for instance, is associated with non‐syndromic ID and does not inactivate the enzyme activity of ST3GAL3 in vitro.^6^, ^7^, ^8^, ^9^ However, when there is a complete loss of enzymatic activity of ST3GAL3, the phenotype may be more severe and present as a DEE.^5^, ^6^, ^8^ Although functional testing was not available for our sibling pair, it is hypothesized that both variants resulted in a lack of ST3GAL3 enzyme activity. It is unclear why our sibling pair had a milder phenotype, given the predicted loss of enzymatic activity in our sibling pair and at present time, precise genotype–phenotype relationships have not been established for *ST3GAL3*‐related DEE. Moreover, there was some subtle phenotypic variability in our sibling pair, with the younger sibling having a less severe motor impairment and systemic involvement, for example. It is possible that unknown genetic or environmental factors could account for this. However, for the most part, sibling pairs have shown similar phenotypes in the literature.^6^, ^7^, ^8^, ^9^

Our work has limitations, including that this is a retrospective case series with few newly described cases. However, this is a rare condition, with limited reports in the literature. There were some missing data the from literature review, and not all cases had all the reported variables of interest available. Given the limited number of cases described in the literature, it is likely that the complete phenotypic spectrum of *ST3GAL3*‐related DEE is unknown.

## 5 CONCLUSION

In summary, we demonstrate that the spectrum of epilepsy in *ST3GAL3*‐related DEE may include early onset infantile seizures in the form of epileptic spasms with evolution to LGS. However, seizures that show a good response to ASMs with prolonged periods of seizure freedom may also occur later in childhood. Furthermore, not all children will be classified with a known epilepsy syndrome. Various seizure types may be observed, including a mixture of generalized and focal seizure types. EEG is invariably abnormal, while neuroimaging is generally normal. However, in distinct cases MRI has shown restricted diffusion and T2 hyperintensity in the brainstem and middle cerebral peduncles. Neurologic comorbidities include ID, motor and language impairment, hypotonia, behavioral disorders, and stereotypies. Tremor may also be uniquely observed. There are limited data to support the use of specific ASMs in *ST3GAL3*‐related DEE and additional larger studies are needed to address this.

## FUNDING INFORMATION

None.

## CONFLICT OF INTEREST STATEMENT

Rajesh RamachandranNair received a research grant from the Ontario Brain Institute and served as a paid consultant to UCB Canada Inc. and Sunovion Pharmaceuticals Canada Inc. Mark Tarnopolsky sits on the Neurogenetics Expert Group for Ontario and the Out of Country Genetics Approval Committee for Ontario. He is the CEO and CSO of Exerkine/Stayabove Nutrition. The remaining authors have no conflicts of interest.

## ETHICAL APPROVAL

We confirm that we have read the Journal's position on issues involved in ethical publication and affirm that this report is consistent with those guidelines.

## Data Availability

The data that supports the findings of this study are available from the corresponding author, upon reasonable request.
